# High visceral-to-subcutaneous fat area ratio is an unfavorable prognostic indicator in patients with uterine sarcoma

**DOI:** 10.1007/s11604-025-01812-7

**Published:** 2025-06-12

**Authors:** Mariko Kurokawa, Wataru Gonoi, Shouhei Hanaoka, Ryo Kurokawa, Shunichi Uehara, Masayoshi Kato, Mizuka Suzuki, Yusuke Toyohara, Yasunobu Takaki, Misako Kusakabe, Nao Kino, Takehiro Tsukazaki, Toshiyuki Unno, Kenbun Sone, Osamu Abe

**Affiliations:** 1 Department of Radiology, Graduate School of Medicine, The University of Tokyo, 7-3-1 Hongo, Bunkyo-ku, Tokyo, 113-8655 Japan; 2 Department of Radiology, Showa General Hospital, Tokyo, Japan; 3 Department of Radiology, Tokyo Metropolitan Cancer and Infectious Diseases Center Komagome Hospital, Tokyo, Japan; 4 Department of Obstetrics and Gynecology, Graduate School of Medicine, The University of Tokyo, Tokyo, Japan; 5 Department of Gynecology, The Cancer Institute Hospital of Japanese Foundation for Cancer Research, Tokyo, Japan; 6 Department of Gynecology, Tokyo Metropolitan Cancer and Infectious Diseases Center Komagome Hospital, Tokyo, Japan; 7 Department of Obstetrics and Gynecology, Showa General Hospital, Tokyo, Japan; 8 Department of Obstetrics and Gynecology, NTT Medical Center Tokyo, Tokyo, Japan

**Keywords:** Sarcoma, Sarcopenia, Low muscle mass, Subcutaneous adiposity, Visceral adiposity

## Abstract

**Purpose:**

Uterine sarcoma is a rare disease whose association with body composition parameters is poorly understood. This study explored the impact of body composition parameters on overall survival with uterine sarcoma.

**Materials and methods:**

This multicenter study included 52 patients with uterine sarcomas treated at three Japanese hospitals between 2007 and 2023. A semi-automatic segmentation program based on deep learning analyzed transaxial CT images at the L3 vertebral level, calculating body composition parameters as follows: area indices (areas divided by height squared) of skeletal muscle, visceral and subcutaneous adipose tissue (SMI, VATI, and SATI, respectively); skeletal muscle density; and the visceral-to-subcutaneous fat area ratio (VSR). The optimal cutoff values for each parameter were calculated using maximally selected rank statistics with several p value approximations. The effects of body composition parameters and clinical data on overall survival (OS) and cancer-specific survival (CSS) were analyzed.

**Results:**

Univariate Cox proportional hazards regression analysis revealed that advanced stage (III–IV) and high VSR were unfavorable prognostic factors for both OS and CSS. Multivariate Cox proportional hazard regression analysis revealed that advanced stage (III–IV) (hazard ratios (HRs), 4.67 for OS and 4.36 for CSS, *p* < 0.01), and high VSR (HRs, 9.36 for OS and 8.22 for CSS, *p* < 0.001) were poor prognostic factors for both OS and CSS. Added values were observed when the VSR was incorporated into the OS and the CSS prediction models.

**Conclusion:**

Increased VSR and tumor stage are significant predictors of poor overall survival in patients with uterine sarcoma.

**Supplementary Information:**

The online version contains supplementary material available at 10.1007/s11604-025-01812-7.

## Introduction

Uterine sarcoma is a rare and aggressive form of malignant mesenchymal neoplasm originating in the muscle and connective tissues of the uterus, accounting for approximately 1% of all female genital tract malignancies and 3%–7% of all uterine malignancies [1]. Despite recent advances in surgical treatment and perioperative care, the risk of recurrence after radical surgery remains high—ranging between 50% and 70%—resulting in a relatively low 5-year survival rate of 52.4%–89.5% [2–4]. The prognostic factors influencing poor outcomes include incomplete resection, advanced stage, and tumor margin involvement [5, 6]. Treatment decision-making in patients with uterine sarcomas is complex as it involves weighing the benefits of surgery, chemotherapy, and radiation against potential complications.

Recent systematic reviews have revealed that assessing body composition parameters such as amount or quality of skeletal muscle and body fat helps predict future mortality in various diseases [7, 8]. Among these parameters, low skeletal muscle mass, frequently measured on medical images and one of the significant sarcopenia indicators, has been reported as a prognostic factor for poor outcomes in patients with various cancers from the head and neck to the gastrointestinal tract and genitourinary organs, including the uterine corpses and cervix [9–15].

In addition, obesity, or a combination of sarcopenia and obesity (sarcopenic obesity), has demonstrated potential prognostic relevance in many types of cancer [16, 17]. In particular, the visceral-to-subcutaneous fat area ratio (VSR) is a promising indicator of body fat distribution that has recently attracted attention. A high VSR indicates poorer outcomes in patients with various types of cancers, such as endometrial cancer, gastric cancer, melanoma, and localized retroperitoneal sarcoma [18–21].

Although body composition parameters have been reported to influence the prognosis of various malignancies, there is a paucity of data on their role in uterine mesenchymal malignancies, such as sarcomas, owing to the rarity of these diseases. Investigating the association between body composition parameters and uterine sarcoma is of critical importance as it may provide valuable insights into prognostic factors and contribute to the development of tailored management strategies for this aggressive tumor type. This study explored the intricate relationship between body composition parameters and uterine sarcoma, focusing on the impact on survival outcomes.

## Materials and methods

### Ethical issues

The institutional review boards approved the present three-center study of the three participating institutions: University of Tokyo Hospital, Bunkyo-ku, Tokyo, Japan (approval number, 2019127NI); Tokyo Metropolitan Cancer and Infectious Diseases Center Komagome Hospital, Bunkyo-ku, Tokyo, Japan (approval number, 2640); and Showa General Hospital, Kodaira City, Tokyo, Japan (approval number, RED255). The requirement for informed consent was waived because of the study’s retrospective nature, and it was clearly stated on the website that patients could opt out of study at any time. All methods adhered to the relevant guidelines and regulations.

### Study population

This retrospective study included all the patients who were surgically diagnosed with uterine sarcomas and underwent both preoperative abdominal CT studies within 4 months before surgery and subsequent surgical treatment and was performed at three Japanese hospitals, including one university hospital and two general hospitals, from April 2007 to June 2023. If any of the clinical information mentioned below or the CT image at the L3 vertebral level was missing, the patient was excluded from the analysis.

### Clinical information

We collected the following clinical parameters upon first admission for surgery: age at diagnosis of uterine sarcoma, body mass index (BMI), histological type of uterine sarcoma (leiomyosarcoma, undifferentiated endometrial sarcoma, endometrial stromal sarcoma, or adenosarcoma), overall survival (OS), cancer-specific survival (CSS), Federation of Gynecology and Obstetrics (FIGO) staging of uterine sarcoma, adjuvant therapy status, and administered regimens [22]. OS was defined as the duration from surgery to death from any cause, whereas CSS was defined as the duration from surgery to cancer-specific death. Clinical information was collected up to August 2023.

### CT scanning and image processing

All patients underwent preoperative body CT scanning for uterine sarcoma. The voltage was held constant at 120 kVp, and the slice thickness was 5 mm. The mean interval between preoperative CT and surgery was 18 days (1–106 days). Most CT examinations were performed in the delayed phase (*n* = 43) with contrast medium administration, whereas the others were non-contrast CT examinations (*n* = 9).

A semi-automatic segmentation program based on deep learning, developed in-house [23, 24], runs on a platform for computer-aided diagnosis research, CIRCUS version 1.6.0 (http://circus-project.net), and was employed to select transaxial images at the L3 vertebral level containing both transverse processes from the pre-surgical body CT images and segment body compositions on the selected plane. Two board-certified radiologists (M.K. and G.W.), blinded to any clinical information, checked all selected CT slices and segmented areas at each slice selected using the semi-automatic segmentation program and amended them if necessary. The following body composition parameters were calculated for each image: skeletal muscle area (SMA, cm^2^), skeletal muscle density (SMD, Hounsfield units), visceral adipose tissue area (VATA, cm^2^), and subcutaneous adipose tissue area (SATA, cm^2^). According to published studies, the density thresholds on CT images are defined as follows: skeletal muscle, –29 to +150 Hounsfield units (HU); visceral adipose tissue, −150 to −50 HU; and subcutaneous adipose tissue, –190 to −30 HU [25–28]. SMI, visceral adipose tissue index (VATI), and subcutaneous adipose tissue index (SATI) were calculated by dividing SMA, VATA, and SATA (cm^2^) by the square of the height (m^2^). VSR (VSR = VATA/SATA) was also calculated. To account for the effects of the contrast medium on body composition parameters (SMA, SMD, SATA, and VATA), adjustments were made using a previously validated methodology [29]. This method standardized the values from non-contrast CT scans to delayed-phase equivalents. Specifically, contrast medium administration in the delayed phase increased SMA by 2.8% and SMD by 7.8 HU while decreasing SATA by 2.9% and VATA by 20.0% [29].

### Determining the optimal cutoffs for each body composition parameter

As there are no standardized cutoffs for body composition parameters in uterine sarcomas, we calculated the optimal cutoffs for each parameter that maximized OS separation using maximally selected rank statistics with several *p* value approximations (MAXSTAT) [30].

### Statistical analysis

We assessed the normality of the distribution for each body composition parameter using Shapiro–Wilk test. The cohort was divided into two groups for each body composition parameter based on the optimal cutoffs and collected clinical data. Survival rates were estimated using the Kaplan–Meier method, while between-group differences (*p* values) were calculated using log-rank test. Cox proportional hazard regression model was used to conduct univariate and multivariate analyses of OS and CSS. Bonferroni’s family-wise correction was applied to variables in the univariable analyses. Furthermore, we investigated the added value of incorporating body composition parameters into prognosis prediction for uterine sarcoma by comparing the concordance index (*C* index) of two models: one based solely on established clinical indicators—such as tumor stage, age, and BMI—and another that integrates these clinical indicators with body composition parameters. The correlation between VSR and BMI was assessed using Pearson’s correlation coefficient. All statistical analyses were performed using R software (version 4.3.1; Foundation for Statistical Computing, Vienna, Austria). Statistical significance was set at *p* < 0.05.

## Results

### Patients’ demographic data

**Fig. 1:**
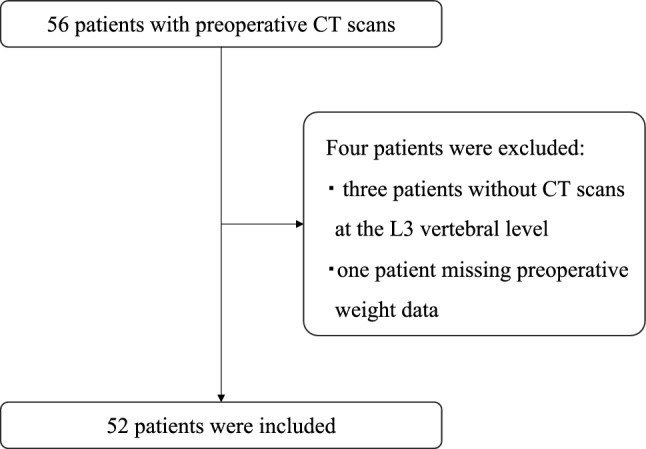
Flow chart of the study

A total of 56 patients underwent preoperative CT scans. They were surgically diagnosed with uterine sarcoma at three hospitals during the study period (21 from the University of Tokyo Hospital, 12 from Showa General Hospital, and 23 from the Tokyo Metropolitan Cancer and Infectious Diseases Center Komagome Hospital). Of these, three patients were excluded because of the absence of preoperative CT scans at the L3 vertebral level, and one patient was excluded because of missing preoperative weight data. Finally, 52 patients were included in the final analysis (Fig. 1). The baseline patient characteristics are summarized in Table 1. The most common histological finding was leiomyosarcoma. Using the optimal cutoff for the present cohort, 82.7% of the patients (*n* = 43) were classified as having low muscle mass. Of 52 patients, 22 (42.3%) died of cancer, and two (3.8%) died of other causes, with a median follow-up period of 16 (interquartile range, 1–134) months. Of the 52 cases, nine were evaluated with non-contrast CT and 43 with the delayed phase of contrast-enhanced CT. A total of 36 patients received adjuvant therapy, and 16 did not. Among those who received adjuvant therapy, the most commonly used regimen was the docetaxel–gemcitabine combination (*n* = 12), followed by a combination of gemcitabine and docetaxel (*n* = 8), and other regimens, such as idarubicin, cytarabine, and Adriamycin.

**Table 1:** Patients’ baseline characteristics

Parameters	Total (*n* = 52)
Age, years, median (IQR)	55 (48.5−61.5)
Histology, no. (%)	
Leiomyosarcoma	37 (71.2)
Undifferentiated endometrial sarcoma	4 (7.7)
Endometrial stromal sarcoma	3 (5.8)
Adenosarcoma	3 (5.8)
Spindle cell sarcoma, not otherwise specified	2 (3.8)
Others*	3 (5.8)
Stage, no. (%)	
I	21 (40.4)
II	3 (5.8)
III	11 (21.2)
IV	17 (32.7)
BMI, kg/m^2^, median (IQR)	21.7 (19.2−24.2)
SMA, cm^2^, median (IQR)	82.1 (69.6−97.9)**
SMI, cm^2^/m^2^, median (IQR)	32.9 (28.1−40.5)**
SMD, Hounsfield unit, median (IQR)	33.3 (33.4−47.8)**
SATA, cm^2^, median (IQR)	78.8 (46.2−133.8)**
SATI, cm^2^/m^2^, median (IQR)	32.7 (19.2−53.3) **
VATA, cm^2^, median (IQR)	37.4 (8.56−69.7)**
VATI, cm^2^/m^2^, median (IQR)	15.7 (3.3−25.8)**
VSR, median (IQR)	0.38 (0.23−0.61)**
Low muscle mass, no. (%)	43 (82.7)

*BMI* body mass index, *IQR* interquartile range, *SMA* skeletal muscle area, *SMI* skeletal muscle index, *SMD* skeletal muscle density, *SATA* subcutaneous adipose tissue area, *SATI* subcutaneous adipose tissue index, *VATA* visceral adipose tissue area, *VATI* visceral adipose tissue index, *VSR* visceral-to-subcutaneous area fat ratio

^*^Other histology included epithelioid smooth muscle tumor and unclassified uterine sarcomas

^**^The values were normalized for delayed-phase contrast-enhanced CT

### Survival discrimination based on optimal cutoff values

**Fig. 2:**
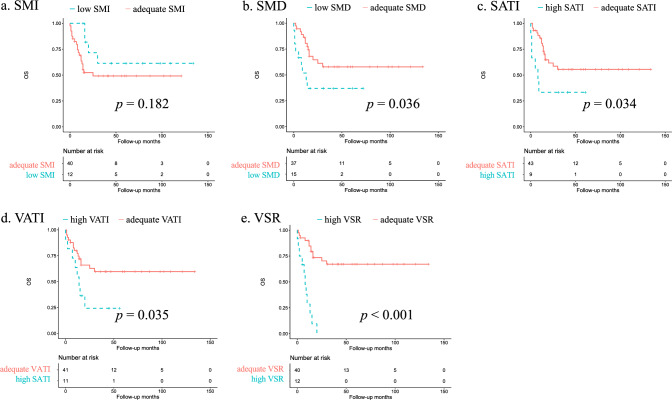
Kaplan−Meier curves for overall survival according to body composition parameters using optimal cutoffs for the present cohort. **A** Skeletal muscle index (SMI), **B** skeletal muscle density (SMD), **C** subcutaneous adipose index (SATI), **D** visceral adipose tissue index (VATI), **E** visceral-to-subcutaneous fat area ratio (VSR)

**Fig. 3:**
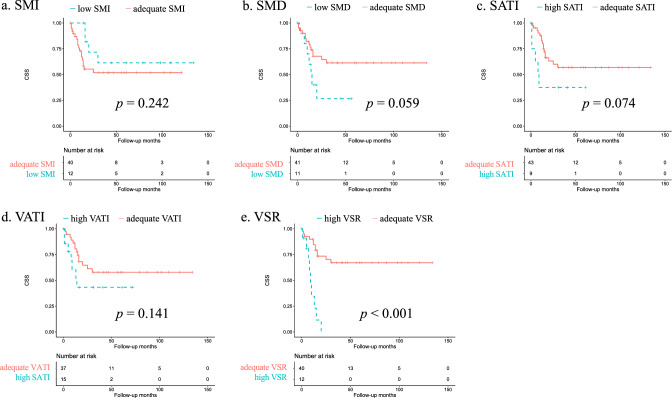
Kaplan−Meier curves for cancer-specific survival according to body composition parameters using optimal cutoffs for the present cohort. **A** Skeletal muscle index (SMI), **B** skeletal muscle density (SMD), **C** subcutaneous adipose index (SATI), **D** visceral adipose tissue index (VATI), **E** visceral-to-subcutaneous fat area ratio (VSR)

The optimal SMI, SMD, SATI, VATI, and VSR cutoffs for the present cohort, normalized for the delayed phase, were 26.3 cm^2^/m^2^, 32.6 HU, 67.6 cm^2^/m^2^, 27.31 cm^2^/m^2^, and 0.73, respectively. Based on non-contrast CT, the cutoffs correspond to 25.5 cm^2^/m^2^ for SMI, 24.8 HU for SMD, 69.6 cm^2^/m^2^ for SATI, and 32.8 cm^2^/m^2^ for VATI. Kaplan–Meier curves depicting OS and CSS based on each cutoff are shown in Figs. 2 and 3, respectively. We additionally assessed the normality of the distribution for each body composition parameter using Shapiro–Wilk test. Among the five parameters analyzed (SMI, SMD, SATI, VATI, and VSR), only SMI was normally distributed. The distributions of the remaining parameters were not normal. The association between the observed distributions and the corresponding cutoff values is also visually depicted (Supplementary Fig. 1). It is noteworthy that VATI exhibited a more pronounced skew than VSR.

### Univariate and multivariate models for OS

In a univariate Cox proportional hazard regression analysis, advanced stage (III or IV), low SMD, high SATI, high VATI, and high VSR showed *p* < 0.05, and after family-wise correction, advanced stage and high VSR survived as candidates for prognostic factors of poorer OS, as shown in Table 2. In a multivariate Cox proportional hazard regression analysis, advanced stage (III or IV) and high VSR were statistically significant poor prognostic variables (Table 2). When combining high VSR and advanced stage, the C index for OS was 0.835 (95% CI 0.766–0.904), which was higher than the C index obtained using only stage (0.693; 95% CI 0.619–0.767). There was a weak positive correlation between BMI and VSR; however, this was not statistically significant (Pearson’s *r* = 0.145, *p* = 0.305).

**Table 2:** Univariate and multivariate Cox proportional hazard analysis for overall survival

Variables	Univariate analysis			Multivariate analysis		
	HR	95% CI	*p* value	HR	95% CI	*p* value
Age≧70 years vs. < 70 years	1.01	0.981–1.04	0.45		−	
Stage III or IV vs. I or II	4.65	1.79–12.06	0.0016**	4.67	1.73−12.7	0.002*
Adjuvant therapy	1.16	0.46−2.95	0.76			
Low SMI	2.09	0.16−1.41	0.18		−	
Low SMD	2.53	1.06−5.99	0.036*		−	
High SATI	2.20	0.81−5.94	0.12		−	
High VATI	2.88	1.18−7.04	0.02*		−	
High VSR	8.90	3.60−22.0	<0.001**	9.36	3.51−25.0	<0.001*

*HR* hazard ratio, *CI* confidence interval, *SMI* skeletal muscle index, *SMD* skeletal muscle density, *VATI* visceral adipose tissue index, *SATI* subcutaneous adipose tissue index, *VSR* visceral-to-subcutaneous fat area ratio

^*^p values below 0.05

^**^Significant after Bonferroni’s family-wise error correction

### Univariate and multivariate models for CSS

Univariate and multivariate Cox proportional hazards analyses showed that advanced stage (III or IV) and high VSR were prognostic factors for poor CSS (Table 3). When comparing the model using only clinical indicators to the model that included clinical indicators plus high VSR, the C index for CSS increased from 0.686 (95% CI 0.606–0.766) to 0.760 (95% CI 0.672–0.848).

**Table 3:** Univariate and multivariate Cox proportional hazard analysis for cancer-specific survival

Variables	Univariate analysis			Multivariate analysis		
	HR	95% CI	*p* value	HR	95% CI	*p* value
Age≧70 years vs. < 70 years	1.02	0.99–1.05	0.268		−	
Stage III or IV vs. I or II	4.28	1.621–11.29	0.003*	4.36	1.59−11.94	0.004
Adjuvant therapy	1.75	0.59−5.23	0.315			
Low SMI	0.52	0.17−1.55	0.24		−	
Low SMD	2.41	0.97−6.02	0.059		−	
High SATI	1.91	0.64−5.69	0.25		−	
High VATI	1.98	0.78− 5.85	0.14		−	
High VSR	7.76	3.01−20.02	<0.001*	8.22	2.96−22.83	<0.001

*HR* hazard ratio, *CI* confidence interval, *SMI* skeletal muscle index, *SMD* skeletal muscle density, *VATI* visceral adipose tissue index, *SATI* subcutaneous adipose tissue index, *VSR* visceral-to-subcutaneous fat area ratio

^*^Significant after Bonferroni’s family-wise error correction

## Discussion

The present study explored several body composition parameters derived from CT images as new prognostic factors for patients with uterine sarcoma. It demonstrated that a high VSR was a valuable prognostic factor for poor OS and CSS, alongside tumor stage. Furthermore, incorporating the body composition parameter VSR into the existing prognostic factor (tumor stage) further increased the predictive capability of OS and CSS.

Obesity is a known risk factor for several malignancies, including uterine cancer [31]. Recent studies have investigated the VSR as a potential prognostic marker [18–20], given that visceral adipose tissue (VAT) is more metabolically active than subcutaneous adipose tissue (SAT) [32]. Our study similarly confirmed that a high VSR is a poor prognostic indicator for patients with uterine sarcoma, highlighting the relevance of body fat distribution in cancer prognosis. Since there are no existing reports investigating the association between uterine sarcoma and body composition parameters, our research could provide valuable insights into how visceral and subcutaneous adipose tissue influence the prognosis of uterine sarcoma and indicate that VSR could help identify high-risk patients with uterine sarcoma.

VAT produces higher levels of pro-inflammatory cytokines like interleukin 6 (IL-6) and tumor necrosis factor-alpha (TNF-α), which contribute to a chronic inflammatory state that facilitates cancer progression by promoting angiogenesis, cancer cell invasiveness, and a supportive tumor microenvironment [32]. Additionally, VAT is associated with insulin resistance and elevated insulin and insulin-like growth factor (IGF) levels, which activate cell proliferation pathways, such as PI3K/AKT and MAPK, thus creating a systemic environment that favors tumor growth [33]. The release of adipokines, such as leptin and adiponectin, further influences tumor biology; leptin, often elevated in high VAT, can promote cancer cell proliferation and migration, whereas adiponectin, which tends to be lower in visceral obesity, has anti-inflammatory and antiproliferative effects [34]. These mechanisms have been suggested in previous studies and may partially explain the observed association between high VSR and poor outcomes although they were not directly evaluated in our study. The anatomical proximity of the VAT to vital organs and its high vascularity may facilitate the spread of cancer cells. As observed in our study, these combined effects may explain why a high VSR is associated with poorer outcomes in patients with uterine sarcomas.

The present study has several limitations. First, it included only a limited number of Japanese patients with uterine sarcoma because uterine sarcoma is a rare malignancy. To address this limitation, we designed a multi-institutional setting. Second, the causal mechanisms connecting a high VSR with poor prognosis in uterine sarcomas remain speculative. Further evidence demonstrating the direct effect strengthens this argument. Third, since there are no widely accepted standardized cutoff values for body composition parameters across rare diseases, such as uterine sarcoma, we used the maximally selected rank statistics (MAXSTAT) method to determine optimal cutoff values for body composition parameters based on OS and then applied these OS-derived thresholds to CSS analyses. Additional research is needed to refine and validate the optimal cutoff values for body composition indices in diverse clinical settings. Fourth, given the pronounced skew of VATI (Supplementary Fig. 1), it is conceivable that VATI may equal or surpass VSR in prognostic performance once examined in larger, more heterogeneous cohorts; it would be desirable for future studies to compare these indices in adequate cohorts.

In conclusion, this study identified a high VSR, along with tumor stage, as a significant prognostic factor of poor overall survival in patients with uterine sarcoma.

## Supplementary Information

Below is the link to the electronic supplementary material.

Supplementary file1 Supplementary Figure 1 The distribution for each body composition parameter. **A** Skeletal muscle index (SMI), **B** Skeletal muscle density (SMD), **C** Subcutaneous adipose index (SATI), **D** Visceral adipose tissue index (VATI), **E** Visceral-to-subcutaneous (PDF 118 KB)
