# Supplementary figures and images for: High visceral-to-subcutaneous fat area ratio is an unfavorable prognostic indicator in patients with uterine sarcoma
Source: Jpn J Radiol. 2025 Jun 12;43(10):1670–7. doi: 10.1007/s11604-025-01812-7 (PMC12479680; doi:10.1007/s11604-025-01812-7)

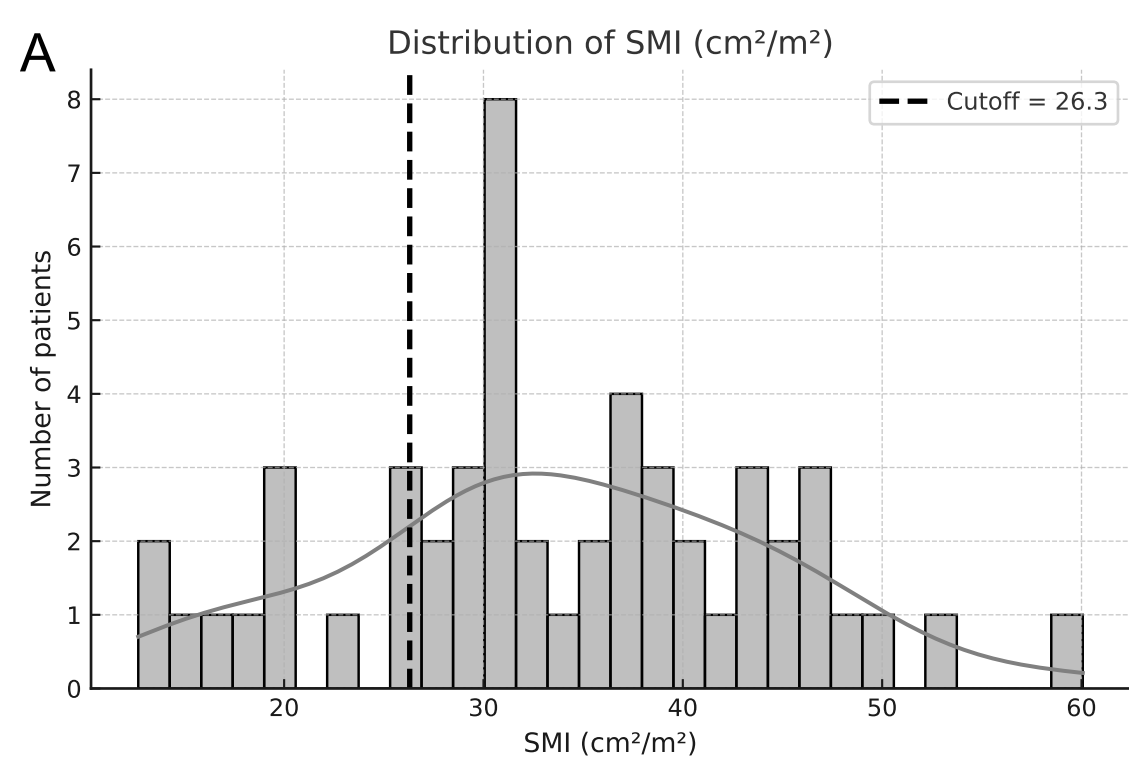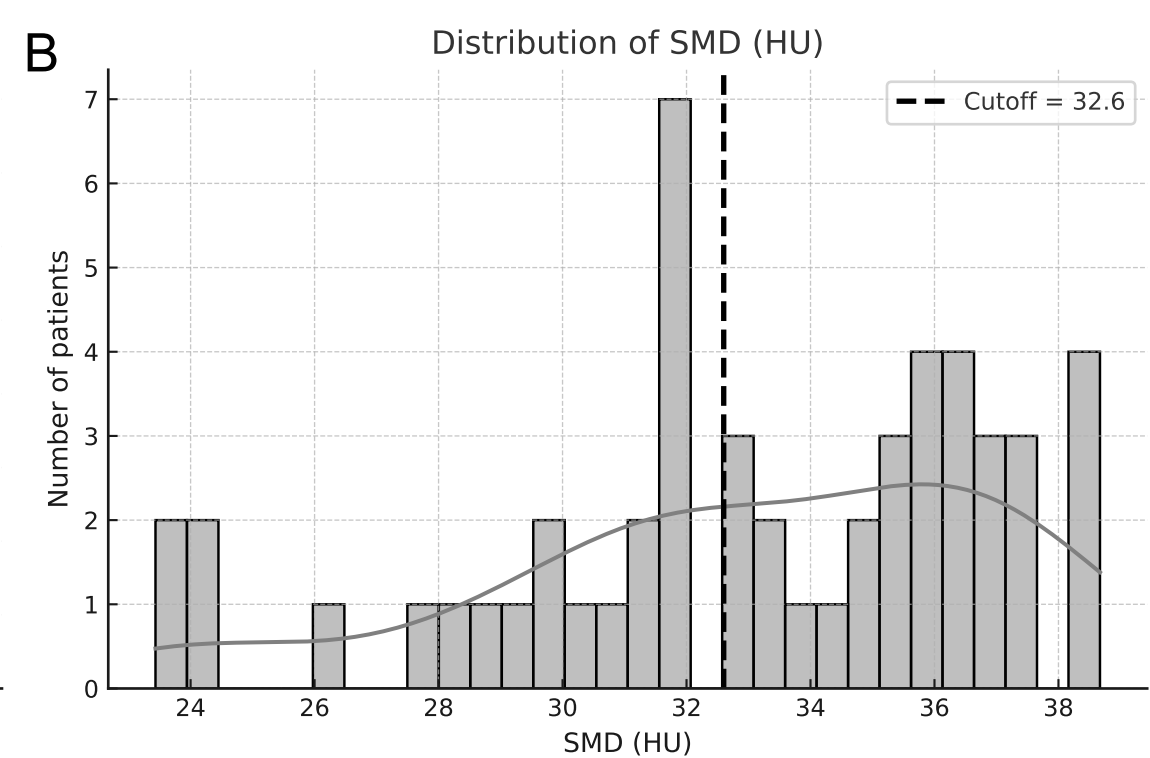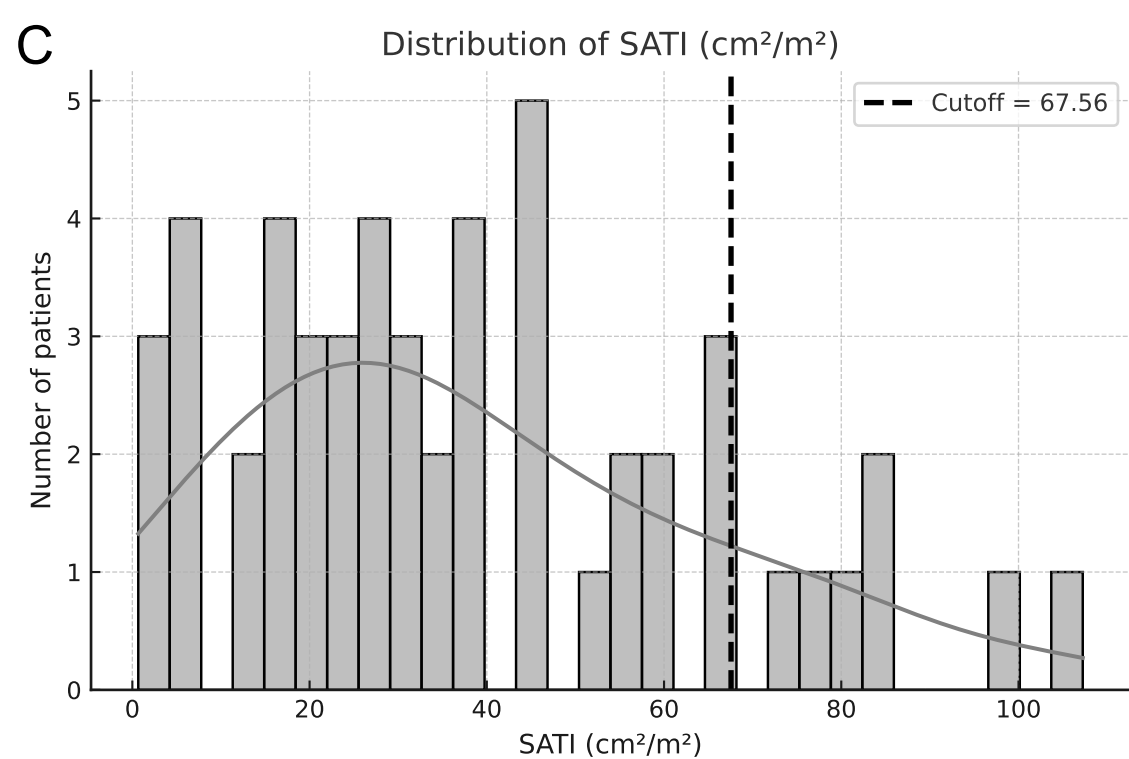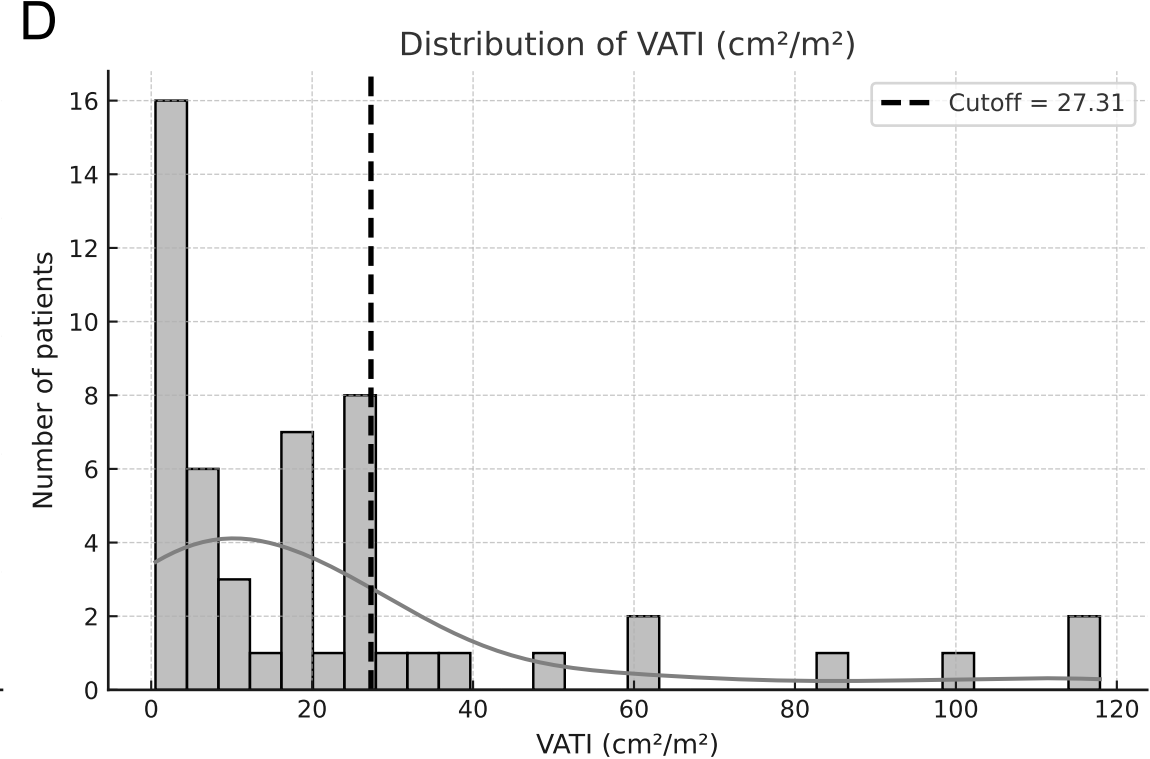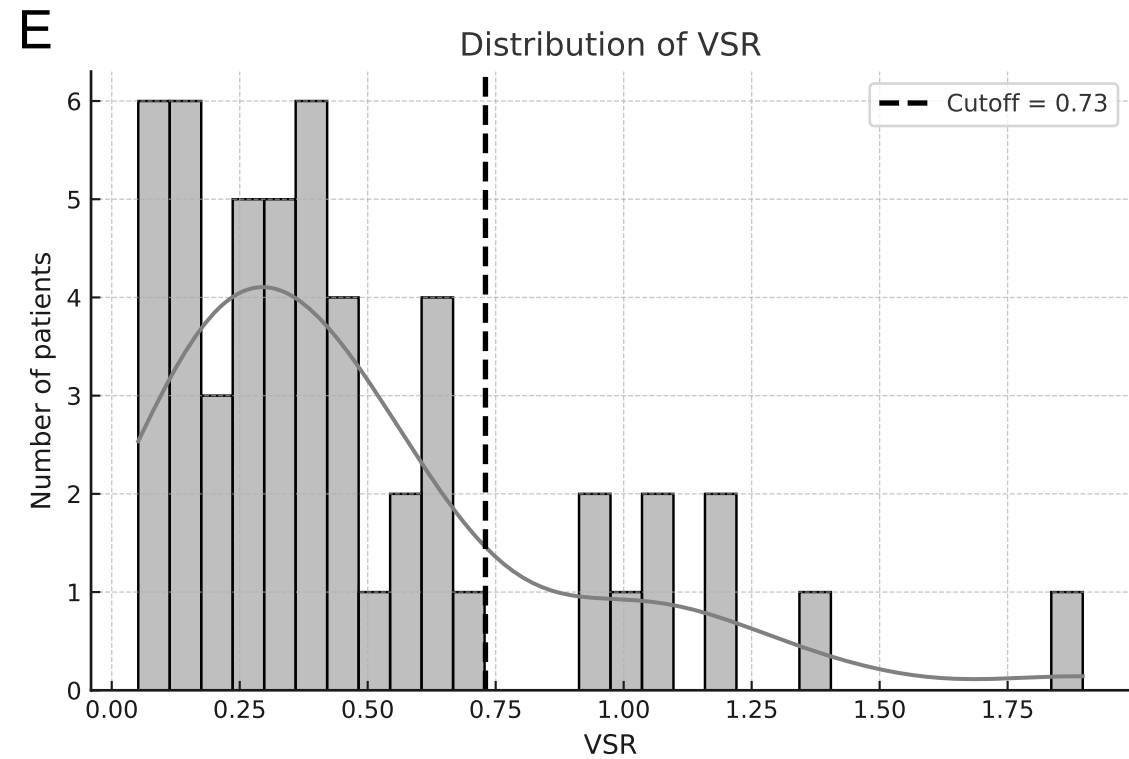

Supplement: Supplementary file 1 — Supplementary file1 Supplementary Figure 1 The distribution for each body composition parameter. A Skeletal muscle index (SMI), B Skeletal muscle density (SMD), C Subcutaneous adipose index (SATI), D Visceral adipose tissue index (VATI), E Visceral-to-subcutaneous (PDF 118 KB) [file 11604_2025_1812_MOESM1_ESM.pdf]
